# A critical realist evaluation of a music therapy intervention in palliative care

**DOI:** 10.1186/s12904-017-0253-5

**Published:** 2017-12-08

**Authors:** Sam Porter, Tracey McConnell, Mike Clarke, Jenny Kirkwood, Naomi Hughes, Lisa Graham-Wisener, Joan Regan, Miriam McKeown, Kerry McGrillen, Joanne Reid

**Affiliations:** 1 Department of Social Sciences and Social Work, Bournemouth University, Poole, Northern Ireland; 2 School of Nursing and Midwifery, Queen’s University Belfast, Belfast, Northern Ireland; 3 School of Medicine, Dentistry and Biomedical Sciences, Queen’s University Belfast, Belfast, Northern Ireland; 4 Every Day Harmony Music Therapy, Belfast, Northern Ireland; 5 Marie Curie Hospice Belfast, Marie Curie, Northern Ireland

## Abstract

**Background:**

Music therapy is increasingly used as an adjunct therapy to support symptom management in palliative care. However, studies to date have paid little attention to the processes that lead to changes in patient outcomes. To fill this gap, we examined the processes and experiences involved in the introduction of music therapy as an adjunct complementary therapy to palliative care in a hospice setting in the United Kingdom (UK).

**Methods:**

Using a realistic evaluation approach, we conducted a qualitative study using a variety of approaches. These consisted of open text answers from patients (*n* = 16) on how music therapy helped meet their needs within one hospice in Northern Ireland, UK. We also conducted three focus groups with a range of palliative care practitioners (seven physicians, seven nursing staff, two social workers and three allied health professionals) to help understand their perspectives on music therapy’s impact on their work setting, and what influences its successful implementation. This was supplemented with an interview with the music therapist delivering the intervention.

**Results:**

Music therapy contains multiple mechanisms that can provide physical, psychological, emotional, expressive, existential and social support. There is also evidence that the hospice context, animated by a holistic approach to healthcare, is an important facilitator of the effects of music therapy. Examination of patients’ responses helped identify specific benefits for different types of patients.

**Conclusions:**

There is a synergy between the therapeutic aims of music therapy and those of palliative care, which appealed to a significant proportion of participants, who perceived it as effective.

**Electronic supplementary material:**

The online version of this article (10.1186/s12904-017-0253-5) contains supplementary material, which is available to authorized users.

## Background

Palliative care services advocate the use of adjunct complementary therapies to address aspects of patient suffering outside the remit of medical science and technology [1].Complementary therapies are recognised in the National Institute for Health and Care Excellence (NICE) guidelines as providing support for adult palliative care patients, and these guidelines highlight the need for evaluation research to determine effectiveness and optimal delivery of such services [2].

Music therapy is a commonly used complementary therapy in hospice services [3]. Music therapy is defined as the use of music in the context of a therapeutic relationship with a professional music therapist to meet the individual’s therapeutic goals [4]. For palliative care patients, these goals include improving their quality of life through the relief of physical and psychological symptoms, supporting communication and easing spiritual or existential conflicts [5].

A small but emerging body of research has identified the benefits of music therapy for palliative care patients, including a Cochrane systematic review and a meta-analysis indicating improved mood and sense of wellbeing, along with reduced nausea, anxiety and depression [6]. This review has been withdrawn by the Cochrane Collaboration because it was out of date. However, it has since been updated by a systematic review using the same protocol, which additionally indicated pain reduction, [7]. However, while studies of music therapy to date have focused on patient outcomes, little attention has been paid to the processes that lead to those outcomes. An understanding of these processes is required if analysis is to go beyond the identification of efficacy in a controlled evaluation setting, so that it can inform policy and practice in terms of the factors that may affect successful and sustainable implementation of music therapy interventions in diverse palliative care settings. Process evaluation using qualitative methods has been recommended for future music therapy trials to aid in identifying factors which contribute to or limit its effectiveness [8].

### Methodological approach

There is a current dearth of strong evidence about the effectiveness of music therapy in palliative care because of the lack of randomised trials [7]. We have therefore initiated a research programme to contribute to the evidence base through a randomised trial [9]. However, such trials on their own are not sufficient if they are concerned solely with comparing the probability of participant’s outcomes. Instead, in order to incorporate process data into the evaluative model, our randomised trial was embedded in a critical realist methodology.

There has been an increasing interest in realist approaches to randomised trials [10–14] to counter some of the identified weaknesses of RCTs, such as their difficulties in generalizing to different contexts, being sensitive to individual characteristics, and discerning the specific effects of components within complex interventions [15–18]. Critical realist randomised trials, while consonant with the procedural steps laid out in the Medical Research Council’s (MRC) framework for evaluating complex healthcare interventions [19], adopt a theoretical position that overcomes some of the tensions inherent in the framework’s attempt to combine traditional assumptions underlying randomised trials with the theoretical bases of the contextual and qualitative approaches that it has introduced [10, 14].

In contrast to the exclusive focus of traditional trials on the relationship between the dependent variable and the experimental variable, critical realism argues that this relationship is the result of underlying generative mechanisms whose powers cause events to occur [20]. The term ‘mechanism’ is used by realists to underline a specific conception of causation which involves the generation of tendencies rather than an invariable relationship between cause and effect. The advantage of taking such an approach is that it opens up the possibility of going beyond identification to explanation, enabling a more nuanced understanding of why causal relationships are rarely constant. In open systems, which typically have a number of mechanisms operating simultaneously, what actually happens will depend on their interaction. Critical realism also asserts that when looking at social phenomena, adequate explanation needs to take account of the fact that the people involved have their own causal powers of interpretation and volition [21].

When applied to healthcare interventions, critical realist evaluation assumes that, in order to explain outcomes, it is necessary to identify how they are influenced by the interaction of the mechanisms contained in the intervention, the mechanisms embedded in the context into which the intervention is introduced, and the responses of the individuals who experience the intervention [22]. Based on these assumptions, critical realist randomised trials incorporate three distinct though interconnected strategies. The first is to enumerate outcomes (which is where randomised trials make their contribution); the second is to develop and test hypotheses about the mechanisms embedded in the intervention and its context; and the third is to uncover how the people involved respond to the resources and restrictions entailed by the intervention and its context [14]. This paper is concerned with the latter two strategies. It builds on a realist review of the literature that sought to develop a theoretical understanding of what was already known about how music therapy works, for whom and in what circumstances [8]. This involves comparing the theories presented in the literature about how and why music therapy can make a difference with the theories of research participants who have had experience of music therapy. The purpose of this comparison is to test, refine or refute existing theories in relation to what works.

Four main theories emerged from our previous critical realist review of the literature about the mechanisms by which music therapy could make a positive contribution to palliative care. Each mechanism was associated with a specific domain of action:

- Within the supportive domain, it can act as a distraction from physical and psychological suffering.
- Within the communicative/expressive (emotional) domain, it can have a cathartic influence, creating a safe channel through which patients can express repressed emotions.
- Within the transformative (spiritual/existential) domain, it can support the search for meaning and transcendence, and can facilitate the creation of a lasting legacy.
- Within the social domain, it can help strengthen social bonds with loved ones.

### Aim

The aim of this study is to examine the processes and experiences involved in the introduction of music therapy as a complementary therapy to palliative care.

### Objectives

- To identify mechanisms contained in the music therapy intervention.
- To compare these with the mechanisms identified in the literature.
- To identify pertinent mechanisms embedded in the social and organisational context.
- To examine the ways in which different individuals respond to music therapy
- To illuminate individual experiences of music therapy of patients, their families and the professionals caring for them.

## Methods

This critical realist evaluation of process, response and experience was undertaken alongside a pilot randomized trial (trail reference number: NCT02791048) testing the feasibility of conducting a definitive phase III randomized trial to evaluate the effects of music therapy for palliative patients in an in-patient hospice setting. The patients in the study fell into two main categories: Those who were admitted to the hospice for symptom management with the expectation of being discharged, and those who were admitted for end-of-life care, and whose life expectancy on admission was short.

Ethics approval was given by the Office of Research Ethics Committee Northern Ireland (ORECNI) -Reference number 16/NI/0058.Informed consent was gained from all participants included in this study. Our UK-based, single-centre, pilot trial involves two parallel groups, one randomly allocated to receive individual music therapy in addition to usual care, and the other allocated to usual care only. Patients in the experimental group receive two music therapy sessions per week lasting up to 60 min each, for 3 consecutive weeks, in addition to usual care. Sessions are tailored to individual patient needs, involving live and recorded music, song composition, life review, active music making, listening to familiar music, and creating legacy recordings. Those in the control group are offered two sessions of music therapy once they have completed their final study visit.

Participants in the randomized trial were recruited via a clinical gatekeeper from an 18-bed specialist palliative care inpatient unit in Northern Ireland. Baseline data collection (before randomisation) included the McGill Quality of Life Questionnaire (MQOL) [23] and socio-demographic data. Follow-up measures were administered at one, three and five weeks [9].

Alongside these quantitative data, qualitative data was gathered from all patients recruited to the study, who provided verbal responses to an open ended question at the end of the MQOL questionnaire on what needs were most important to them. If they had received music therapy they were also asked if it helped meet their needs and if so in what way. Patient data presented in this study were collected at baseline, one, three and five week follow-up. Sixteen patients recruited between June 2016 until February 2017 and who received music therapy were included in the study.

A purposive sampling approach was used to recruit practitioners from the same inpatient unit. Potential participants (those who had worked at the hospice since the music therapy study had commenced) were contacted via a clinical gatekeeper who invited them to take part in a focus group. Focus groups with physicians, nursing staff, social workers and allied health professionals (AHPs) were undertaken to elicit their theories of what helped or hindered the implementation of music therapy within the hospice setting on the basis of their experience of the music therapy intervention.This was supplemented with an interview with the music therapist delivering the intervention (see Additonal file 1 and 2 for focus group and interview schedules).

Three focus groups were conducted, one involving seven physicians, one involving seven nurses, and one involving two social workers and three AHPs. A purposeful sampling approach was used to recruit Health Care Practitioners (HCP) with a direct patient role, approximately two months after trial initiation to ensure they had experience of music therapy within the hospice setting. It should be noted that HCPs had indirect experience of music therapy through awareness of the music therapist’s presence within the inpatient unit and through patient, family/carer self-reports. The sample size was dictated by data saturation, in that no significant novel information was generated by the third focus group. A semi-structured focus group guide was developed to elicit practitioners’ theories about how music therapy works, for whom, and in what circumstances. Each focus group lasted between 30 to 45 min and took place within a private room at the hospice. TM, an experienced qualitative researcher conducted the focus groups and the interview. Data were collected during December 2016 to January 2017.

### Analysis

Focus groups and the interview were audio recorded, transcribed verbatim by an independent transcriber, and checked for accuracy by TM. Transcripts and open responses from the quantitative questionnaires were analysed using a thematic analysis based on Newell and Burnard’s framework [24]. Key points highlighted by participants were arranged by TM into themes and sub-themes in relation to the theoretical framework developed previously from our realist review of the literature [8] in order to further test, refine or refute existing theories in relation to what works, for whom and in what circumstances. The analysis was triangulated by SP on the basis of one randomly selected full focus group transcript. A random selection of analysis was examined by SP, followed by discussion and the seeking of consensus to validate key themes and sub-themes. As TM and SP conducted the previous critical realist review on music therapy for palliative care [8], a third author (JR) with no previous music therapy involvement validated the final analysis and interpretation. Data analysis was further confirmed by the music therapist and an AHP. The same data analysis procedures were used for the open text respondents of patients, although, because this arm of the research elicited less data, we cannot claim to have reached the point of saturation.

The data presented here is entirely qualitative. As such, the knowledge claims of this study are those of qualitative research, which aims at the in-depth illumination of the experiences and interpretations of its participants [25]. In this case, it provides an insight into the at times extremely poignant individual perspectives of the effects of music therapy. In doing so, it adds to the evidence base for the use of music therapy in palliative care. However, as with all qualitative research, the issue of the data’s reliability as a guide for clinical practice is problematic (26).

A realist approach to research validity was used [26]. This takes into account the different perspectives of the stakeholders involved, while at the same time asserting that those perspectives relate to real entities. It therefore rejects the aesthetic and rhetorical criteria frequently used in qualitative research in favour of a more traditional approach that regards validity as the extent to which research reflects accurately that to which it refers. From such a methodological perspective, criteria such as transparency, accuracy and utility can be used to enhance the level of confidence practitioners have that the findings presented accurately portray and explain the issues being addressed, and will inform their practice [26]. Transparency was enhanced by thick description of practitioners’ perspectives [27]. A reflexive journal was used throughout data collection and analysis to record decisions and reasons for them. However, while these approaches enhanced the validity and reliability of the research, we accept that their reliability in terms of generalizable clinical outcomes remains provisional until they are triangulated with the results of a randomised trial.

## Results

This results section examines, in turn, mechanisms identified within the therapeutic programme, mechanisms identified as embedded in the organisational and cultural context within which the programme was introduced, and the responses to and experiences of the intervention of those affected by it.

### Therapeutic programme mechanisms

This section identifies the mechanisms that participants perceive to be contained in the music therapy intervention. As previously noted, this section is structured around a theoretical framework developed in our earlier realist review of the literature on music therapy for palliative care. This framework includes the supportive, communicative/expressive, transformative, and social domains.

#### Supportive (physical and psychological domain)

Candidate theories from the literature propose that music therapy acts as a distraction from physical and psychological suffering [8]. This was supported by our findings:

*I think it’s a distraction for some people. So instead of focusing on the illness, they’re focusing on music and something else that’s enjoyable, so it’s a positive for them and takes them away for a period of time to that other place, and actually, another patient said that to me, that that’s what it did. So, for that period of time that they had the music therapy, they did not think about their illness at all. (AHP 1)*

However, this provides a rather simplistic explanation of how music therapy works, and findings from this study help refine our understanding. For example, practitioners saw music therapy as helping individuals reframe their identities from ‘patients’ to people with unique pasts, interests and personalities.

*They don’t see it (music therapy) as therapy. They see it as something enjoyable and about something other than their bowels and their pain. It treats them as a human with interests and loves and dislikes and a past, as opposed to, okay, let’s just discuss how medication is helping them today. (Physician 1)*

*I think it can be a by-product, distraction, but it’s (music therapy) very much about them as a person, their personality, their choices, their family and a focus on them and living. Reminiscing has been a huge part of sessions, and like I’ve said, it’s not all about music in the sessions. Sometimes it’s about me listening to them, and about all the things they have done in their life. (Music Therapist)*

The therapeutic mechanisms generated by music therapy appeared to be its ability to help patients reconnect with happier memories, to identify key moments in their lives that helped define their important relationships, and to help them have fun again. For example, practitioners reported how song choices were often surprising to them because of their upbeat nature. The music therapist also reported how much enjoyment patients had playing the instruments, even those who did not see themselves as musical. Not only did this give patients a sense of achievement, it also helped bring out the playful side of their personality, which they were able to share with partners and extended family.

*Sometimes the cameras come out and they take videos if there are a lot of instruments involved and there are songs that they associate with happy memories as a family. They would record this as well, especially if they see their loved one playing an instrument and they never thought this would happen, especially not in the hospice or anytime throughout their life, and they’re having fun together. (Music Therapist)*

Patients reported that music therapy helped them to relax, which in turn helped lift their mood. They found the music therapy sessions gave them something to look forward to, lifted their spirits and provided a ‘fun’ space for them and their families/friends which improved their emotional wellbeing.

*Sitting listening to her (the music therapist) sing and play music helped me feel relaxed. It lifts you. You [are] normally sitting moping but it takes my mind off the monkey (cancer) and gives me something to focus on. (Patient 4)*

While intuitively clear to practising music therapists, patient reports further highlighted that the music alone was not the key therapeutic resource, but that the music therapist in combination with the music was central to meeting therapeutic outcomes. The key therapeutic mechanism appeared to be the relationship between the patient and music therapist. This is consonant with the music therapist’s primary aim at the start of the therapeutic process, which is to facilitate clinical goals for each patient. Patients reported feeling a deep connection with the music therapist that surpassed the expectations they had of the therapy. The act of sharing and creating the musical experience together appeared to strengthen this connection, along with the therapist’s ability to help them feel listened to, be comfortable with themselves and to have fun.

*With music therapy it’s all about building up that relationship, through creativity and giving that alternative experience. The clinical aims could be relaxation, it could be legacy, it could be pain management, alleviating anxiety, working with families and making new memories for them. But it very much depends on the client and the client-led approach. (Music Therapist)*

*I found the music therapist really helpful and caring. She talks away and asks how I’m feeling and plays different music to help lift my mood, and it does. She’s very jolly and picks happy music that lifts my spirits. (Patient 5)*

#### Communicative/expressive (emotional domain)

According to the literature, one of music therapy’s key therapeutic mechanisms is its cathartic influence in relation to creating a safe channel through which patients can express repressed emotions, which if left unresolved can intensify their physical and psychological suffering [28]. Our findings supported this assertion, with both practitioners and patients reporting on how music therapy helped patients express themselves in a way they never thought they could.

*Being able to tell my story about my children, how I feel about them, how proud I am of them, to be your mum. Music therapy helps me put it on paper, expressing my love for them, my legacy of love for them. Gives me comfort to know they will have something to go back to (Patient 4)*

*Another patient wrote lyrics based on her family, and then the music therapist put that to song. So that lady had said, “This is my thank-you to my family and this is how I want to express myself”. It was difficult putting those things down into words, but she found a way to express herself through the music and she said… “I don’t know if I would be able to say these things, but because it’s put down in a song…” (Physician 5)*

Music therapy also aided communication between patients and practitioners.Rather than having conversations focused largely on medical care, music therapy appeared to encourage a more personal avenue of conversation with practitioners around their background and loved ones., This also appeared to help facilitate patient-centred care in terms of knowing the patient’s preferences, needs and values.

*It can tell you a lot about a patient because of their choice of songs. You’ve got those who like religious music, hymns and things ... And others will choose like the Beatles songs and different things like that. You can tell a lot about a person and their background… (Nurse 2)*

*Sometimes it opens up a conversation as well. So, a particular lady, who made a song, she brought up sometimes, if she’d had that session, and she brought up then talking about her children. (Physician 6)*

Music therapy could also provide emotional comfort for patients’ loved ones through seeing them have fun, feeling relaxed, and at peace during a very difficult period in their life. As an earlier quote by the music therapist demonstrated, family enjoyed capturing these happy moments on camera or video. Patients also reported their loved ones finding comfort from sharing their music therapy sessions.

*My husband has been present [at the music therapy sessions] and gave lots of suggestions, like the music of our first dance at our wedding. He really enjoyed it and it really lifted his spirits too. (Patient 5)*

*My husband thinks it (music therapy) is a bright spark in a dark environment. (Patient 2)*

#### Transformative (spiritual/existential domain)

The search for meaning, transcendence, creating a lasting legacy, and the comfort that this legacy could provide to both patients and their families were the key therapeutic mechanisms identified in the literature [8]. Our findings in this study supported all these theories and shed further light on how music therapy mechanisms operate. Practitioners and patients reported how music therapy had a way of helping patients surpass their current position and find peace.

*Music therapy helped me get onto that higher plane and transcend what I’m dealing with. (Patient 7)*

Our findings also supported the theory that music therapy could help patients reinforce their sense of meaningfulness.

*Music therapy lifted me. Making the legacy CD helped me to see my life has been very worthwhile. (Patient 1)*

#### Social domain

A key therapeutic mechanism identified in the literature was music therapy’s ability to strengthen social bonds with loved ones [29, 30]. This was supported by our findings in this study, as evidenced by practitioner reports.

*One of my patients used it as a time – her husband was always there and involved, but she valued time with her sister, so it was kind of their time together - that was separate and ringfenced off from anything medical or anything about getting better or getting stronger. It was entirely relationship-building, and they would sing songs together, and it was just such a joy for them. So, I think it really…confirmed that relationship and probably strengthened it at the end of her life as well. (Physician 7)*

In addition to music therapy strengthening intimate bonds, the products of therapy, in the form of outputs from legacy work, were also regarded as powerful mechanisms for facilitating loving communication and ongoing connections after the death of a loved one.

*One of our patients, like he never would have spoken about feelings and stuff, and like I mean he composed a whole song for his wife and literally handed her the CD and was like “Listen to that on the way home”, and she was just like “That’s the nicest thing you’ve ever done for me”, and it was all about her, so it was lovely. (AHP 5)*

*Legacy work really gives them (patients) a sense of purpose and captures the person’s personality. Their loved ones can listen to it (songs written by patients) and remember their character and possibly help them through the grieving process. And for relatives to know this was made for them to listen to after they (loved one) had passed away. They are still connecting with them. (Music Therapist)*

Other social mechanisms identified in the literature included creating a sense of community within the setting, which was again supported by our findings in this study. Practitioners reported how music therapy helped reduce the isolation patients could experience from being in single rooms:

*The patients here are quite isolated because they’re in single rooms. They don’t tend to mix as a group, and yet, something like that, it draws them out into a more communal setting. There’s something that goes on outside the room for them. (Nurse 5)*

Practitioners felt the provision of music therapy and its positive impact on not only patients but also staff working in the clinical area helped humanise the hospice setting, had a calming effect and added a sense of pleasure to what could often be a very sad environment. Although sessions took place in patient’s rooms, some of the rooms led out into the hospice garden where patients would sometimes choose to have their session.

*Well, I just think of XX (music therapist) in the summer when she was out in the garden…all of our rooms then just on that side, everybody heard, so we all benefited from the music therapy in relation to that. It’s very calming I thought. We’ve all benefited from it. Just had that lovely…a nice atmosphere… (AHP 4)*

Staff also reported on the indirect benefits that music therapy had on them, from hearing it and seeing the benefit to their patients.

*I do feel it’s had an impact personally and professionally because it lifts you out of…sometimes the spiral of sadness that you can…go into here, and it just is a reminder [half-laughs] of…just…something maybe a wee bit more uplifting and happy. I know that sounds really simple, but that’s how I’d describe that. (AHP 2)*

### Contextual mechanisms

The critical realist review of the literature [8] identified a number of contextual mechanisms that appeared to generate support for music therapy implementation. These included organisational support, protected time and space for music therapy sessions to take place, and staff support for the music therapist [8]. An understanding of the aims and a belief in the benefits of music therapy for patients also appeared to be important for generating support within an organisation [8].

Our findings supported all these theories such as the importance of organisational support for music therapy in the form of protected time and space for sessions to take place, and cultural support in terms of attitudes towards the music therapist.

*We are respectful of when she’s (the music therapist) with that patient and we let them have the time for the music therapy. (Nurse 6)*

The staff felt that having an understanding of what music therapy involved was an important factor in their acceptance of it as a valid component of care.

*Yeah, that was what was good (being aware of what music therapy is), because she came and she told us what it all about, and actually, that first couple of weeks, when we saw her on the ward… you then just got very familiar with the types of things that she did. (AHP 1)*

Our findings also suggested that first hand reports to clinicians from patients and families reinforced their belief in the effectiveness of music therapy and hence their support for it as a significant therapeutic intervention

*So you would go into a room and somebody would say to you “No, I’ve got my music therapy”. …wouldn’t miss it for the world, so sent me off [laughing] until they’d done their music, so that’s how important it was to people! (AHP 1)*

*I know some patients, when they knew that XX (music therapist) was coming, you could see them brightening up and looking forward to a visit. (Nurse 3)*

An important aspect of cultural context is practitioners’ perceptions of the role of adjunct complementary therapies in healthcare. Palliative care has traditionally had a more inclusive culture than some other clinical specialities, and this was reflected in our findings that it enabled a more holistic therapeutic armamentarium.

*It’s another thing on our portfolio that we could offer patients, so it’s a positive thing to happen in the unit. I think it has added value. I think, if we’re seeing patients enjoying something, it’s good for everybody. (Physician 4)*

Some clinical respondents went further in terms of their perception of the importance of music therapy to end of life care:

*As far as I can see, the staff have been very supportive of the music therapy, you know, because, it’s just like, oh, no, you can’t interrupt in that room because they’re having the music therapy – it’s being seen as a vital part of their treatment. (Nurse 6)*

Findings from this study also supported those in the literature indicating that music therapists should be part of the multidisciplinary team (MDT) [29, 31]. However, one practitioner felt that while music therapists should be seen as part of the overall care team, their time would be best spent with patients rather than at MDT meetings (MDMs).

*I don’t think it would be best use of their time (the music therapist) to sit through two MDMs. I think they’re better used seeing patients, and as long as they liaise with one member of staff, they can then pass it on. (Physician 3)*

It is not possible from these data to ascertain whether this was a functional response to the limited time that music therapists had in the hospice, or an exercise in professional boundary maintenance. However, given the overall support for music therapy within the setting, it appears that hospice practitioners were sensitive to the limited time music therapists had with patients. For example, practitioners would liaise with the music therapist in relation to patients’ needs; a core aspect of MDT working within the hospice setting.

*Very often I’d be getting my hand over I would find out a little bit about the patient, and what their needs might be? (Music Therapist)*

Our findings have allowed us to identify an additional theory related to the importance of flexibility when delivering music therapy to palliative care patients. In this context, flexibility refers to having a more flexible approach to *timing* of sessions rather than flexibility of approaches used within the music therapy session which is standard practice. For example, sessions were structured around the music therapist’s availability, which was limited by the amount of research funding available for the intervention in the randomised trial. Practitioners felt that more flexibility was required in terms of sessions in response to the unpredictability of patients’ disease trajectory.

*I think you need to be a bit more flexible with the availability of sessions and things to suit the patients. It’s just a little bit more unpredictable how the patient is going to be on the day. If I was only here on Tuesday and Thursday afternoon, I wouldn’t be able to see all of my patients. I’d only be able to see maybe half of them. The appointment system maybe works for some patients but not all. (AHP 5)*

The inflexible timing of sessions indicates a key barrier not raised previously in the literature, but highlighted in the findings of this study related to resources. While all practitioners saw the value of music therapy to the overall care of their patients, they also recognised that funding was the key barrier to implementing music therapy within the hospice setting.

*As long as someone’s going to pay for one (a music therapist), I can’t see there being an issue. It’s funding, isn’t it? (AHP 5)*

### Responses and experiences of participants

The literature examined in our realist literature review did not help identify theories in relation to whether responses to music therapy might differ according to the characteristics of patients, as those earlier studies provided no information on who was more likely to take up music therapy or specific benefits for different types of patients. However, our findings in this study do help shed more light on this underdeveloped aspect of the process. While there was overall consensus from practitioners’ perspectives that benefits of music therapy were universal, differences did emerge in relation to specific benefits for different types of patients. For example, practitioners recognised that while the music therapy could be beneficial irrespective of the patient’s diagnosis, gender or age, it was also flexible enough to cater to patients’ individual needs.

*It’s across the board, across the age spectrum, because XX (music therapist) was able to cater for any taste and actually respond to the person’s need (AHP 4)*

*I think music is universal, and different types of music will appeal to different people, but in everybody’s life, there has been some significant music. I’m not sure that we could say it only works for certain types because, you don’t have to be active, you don’t have to be a high-performance status to enjoy it. You know, enjoyment is a very individual thing, so, in that sense, it makes it a fairly universal option. (Physician 4)*

However, it is important to note that while practitioners’ often viewed ‘fun’ as a key outcome of music therapy, other outcomes, such as expressing repressed emotions better represent key clinical goals for the music therapist.

*Fun is a by-product and that’s why it’s so important for staff to know what music therapy is about because they hear the music through the wall and ‘oh that sounds like fun’ and it usually is fun, but the other things are the important things. I will be thinking is this what this person needs, maybe we need to focus on these things that they’re repressing. Like after playing a song and they become really emotional, I’ll be there to support them and help them work through the underlying why are these words so significant for you? (Music Therapist)*

Respondents also alluded to certain types of patient who may derive specific benefits, such as those whose religious beliefs were very important to them. For patients whose faith was a great comfort to them, music therapy appeared to help them strengthen that faith and continue spiritual practices while in the hospice setting.

*A lot of the kind of ones I suppose who have sort of a religious faith seem to get a lot from it because I suppose they’re maybe not getting to church and stuff on a Sunday and they would get so much from that (AHP 5)*

*Music therapy helped me with praise and worship which raised me up. Helped me get onto that higher plane and transcend what I’m dealing with. (Patient7)*

Practitioners discussed how patients who were more introvert about their feelings also seemed to benefit. The expressive avenue of music therapy was seen by respondents to ease psychological anguish.

*And it’s not so much about who might benefit the most, but from a kind of a clinical perspective, the people that I’ve really wanted to access it for before are the people who don’t like talking, or they feel themselves “I’m not a good talker” so for people who just…failing to engage them on a kind of…an emotional level or about their psychological needs…that it can be useful for easing their suffering. (Physician 4)*

Those who had a longer prognosis appeared to benefit from legacy work and song writing that gave them an alternative focus between music therapy sessions.

*I think the patients who were with us for a longer time – they had time to write a song or they tend to delve into it beyond the superficial. There’s the other layers of therapy (legacy work and song writing) that a patient with a longer prognosis got to benefit from. (Physician 1)*

In relation to who felt the effects of music therapy, it has already been noted that both patients and clinicians reported its benefits for them. Clinicians also noted that family and carers reported benefits from music therapy in terms of lightening their emotional despondency. For some families, music therapy also helped facilitate closure by providing a safe space to say their goodbyes.

*One of the patients… very shortly before the patient died, the music therapist had… found out what childhood songs they’d all sung together, and the whole family all sang these songs together, and they all described it as being hugely emotional, and that was almost like a way of…saying goodbye. So, that was very powerful for that particular family. (Physician 6)*

Some practitioners also shared the assumption that this variant of music therapy would have sustained effect, especially for the children of terminally ill patients:

*And I think with legacy-building as well, for their kids to have that in the future, the story of their life together, it’s pretty amazing to have that in the future… (Physician 6)*

Only a small percentage of patients who were eligible to take part in the pilot study declined citing “*I have too much going on*” as a key reason. This reasoning may be generated by the hospice setting itself in relation to the many clinical activities that take place.

*the ones who said they didn’t want it... can find it (the hospice setting) a little tiring. It is a very busy day, in a hospital or a hospice. Your door opens and you’ve no control over it. (Physician 3)*

## Discussion

The results of this critical realist evaluation are encouraging, in that they indicate that at all three levels of analysis – therapeutic programme mechanisms, contextual mechanisms, and people’s responses to these - there are reasons to be confident that music therapy conducted within the context of hospice care can be effective in improving the lives of those recipients of end-of-life care who are receptive to it, along with their loved ones. While we acknowledge that the majority of our findings are not novel, and have been cited in the extensive previous knowledge base on music therapy within palliative care, they provide a more nuanced picture of what works, for whom and in what circumstances; in line with the critical realist evaluation approach.

### Therapeutic programme mechanisms

Data from the evaluation supported the findings of the realist review (8) concerning therapeutic mechanisms, providing a richer analysis of those mechanisms than has previously been available. In terms of supportive mechanisms, our results indicate that potential benefits extend beyond the capacity of music therapy to provide a temporary distraction from the effects of illness [32–35] and also offer participants the opportunity to transcend in a more fundamental way their physical difficulties and maintain their identities as rounded human beings rather than just patients. The opportunity to relax [35] can also reduce patients’ pain and anxiety. Music therapy also provides patients and their families with the opportunity to have fun [35], despite their challenging circumstances. The creative activities that the music therapist designs and offers provides patients and their families with the possibility of expressing repressed emotions [29, 36–38] in the context of a strong therapeutic relationship [39] and the dedicated time to develop it.

In terms of expressive mechanisms, our results add to those of the literature, indicating that music therapy can provide a vehicle through which patients can express repressed emotions [29, 36–38]. Music therapy also opens up the opportunity to enhance patients’ communication with staff, which in turn can facilitate more holistic care. This finding highlights the vital contribution music therapy can have as an adjunct treatment option in light of previous research showing that enhanced communication and holistic care are rated as essential components of good palliative care by patients and their family/carers [40]. Positively contributing to the current knowledge base, our study also confirms that a patient’s use of music therapy to express themselves can provide comfort to their loved ones [39, 41, 42].

In relation to transformative mechanisms, our findings reinforce those of the literature that music therapy can provide mechanisms for patients to transcend their situation, find meaning in their lives, and be more at peace [35, 42, 43].

Turning to social mechanisms, our findings support those in the literature that a key mechanism was the capacity of music therapy to strengthen bonds between patients and their loved ones [37]. Our study adds to this body of knowledge by identifying legacy work as a key mechanism [43, 44]. In addition, our findings indicate that the scope of the social influence stretched beyond intimate relationships, and also had the capacity to promote a sense of community within the hospice [44] and help further humanise this setting.

Hospice staff also reported that the addition of music to the environment combined with the benefits that they felt patients were gaining from this form of therapy, provided staff with an emotional fillip [45].

Finally the crucial role of music therapists in the effectiveness of the therapy needs to be noted [39]. Although both practitioner and patient’s perceptions of the therapeutic mechanisms of music therapy appear to focus heavily on ‘music’ as having inherent properties to generate the beneficial outcomes observed, it is important to recognise the music therapist’s agency in all of this. Music therapists adopt an array of approaches designed to generate therapeutic outcomes based on the individual patient’s clinical needs. They plan sessions and adapt their implementation to achieve specific clinical goals for patients. Key to this is the establishment of a therapeutic relationship through the use of shared musical experiences. The therapeutic relationship is further strengthened by creating improvised music or songwriting together. The shared creativity that this entails helps explain why such a strong relationship is established between the music therapist and their patients [28].

### Contextual mechanisms

Reflecting the requirements for effective implementation of music therapy identified in the literature [32, 45, 46], our results demonstrated that, for the most part, hospice setting and culture were conducive to the effects of music therapy. Organisational support meant that music therapists were provided with the appropriate time and space to perform their therapeutic activities. In addition, education of staff about the nature and function of music therapy, combined with reports of patients and relatives to staff concerning its beneficial effects meant that there was a strong culture of support.

Another novel contextual factor identified from this study as limiting music therapy’s effectiveness was the lack of flexibility in scheduling delivery. Rather than therapy being performed at times when patients were most receptive or able, therapeutic sessions were limited to the two three-hour windows per week that the music therapist was available. Staff accepted that the degree of flexibility that could be offered was a direct function of the financial resources available to the hospice to fund music therapy, indicating a key contextual mechanism influencing the effectiveness of therapeutic mechanisms.

### Responses and experiences of participants

Consonant with the literature, our findings did not identify any general demographic factors that appeared to influence people’s willingness to engage in music therapy, with staff reporting that those who took part were demographically heterogeneous in terms of age, gender, and musical background.

A novel finding of this research was the identification of specific benefits for different types of patients. Those who were religious tended to use it to celebrate and strengthen their faith; those who had difficulty in expressing their feelings used it as a vehicle to open up emotionally, and those who had sufficient time left to engage in the creation of a musical legacy found that beneficial.

Another heartening finding related to practitioner and patient reports that the loved ones of those patients who decided to avail of music therapy gained considerable emotional and relational benefits. We did not have any reports from relatives of participants that they found music therapy detrimental to their quality of life. However, we cannot rule out the possibility that relatives who felt any detrimental effects simply did not voice their concerns.

However, it should be noted that not all patients were convinced that music therapy would be beneficial to them. Music therapy takes time and energy, and at end-of-life both these patient resources are in short supply. As a result, people may choose to use these precious resources in other ways that they feel more beneficial. This indicates that it is not appropriate to regard music therapy as a universal therapeutic regime in end-of-life care.

### Limitations and strengths

The main strength of this research is its illumination of the processes that lie behind the clinical outcomes of music therapy, as reported by staff and participants. This helps to explain how, in what circumstances, and to whom music therapy can be effective in improving quality of life of those nearing the end of their life. However, while providing an important addition to the evidence base concerning music therapy, these results cannot be regarded as definitive for at least two reasons.

First, they report on the findings from a study in a single hospice. This is especially pertinent in relation to contextual mechanisms. For example, the Irish cultural context in which the research was conducted, with its putative valorisation of music making and communally-oriented approaches to death and dying, may have reinforced the effects of music therapy in a way that might not occur in other cultural contexts. Further comparative critical realist evaluations in different palliative care contexts are required to ascertain the degree to which these results are generalizable and the degree to which they are specific.

Second, while the results provide rich evidence about the processes of and responses to music therapy, by themselves they do not allow us to draw definitive unbiased conclusions about its clinical effectiveness compared to the absence of music therapy or other interventions in this setting.

Thirdly, while all eligible practitioners were invited to participate in this study, those who took part may represent a biased sample of practitioners who support music therapy. We cannot rule out the possibility that those who did not take part had less positive opinions on music therapy within the setting.

## Conclusion

Probably the most significant aspect of this research is its establishment of a synergistic relationship between the mechanisms contained in music therapy and those inherent in the hospice context, which in turn were seen to appeal to a significant proportion of participants. Within these general parameters, at least two specific findings suggest ways to enhance the experience of those choosing to undertake music therapy.

The first relates to the articulation between therapeutic mechanisms and people’s responses to the intervention. The study established that music therapy contains a multiplicity of mechanisms that operate in different ways. Correspondingly, respondents who engaged with music therapy chose to be receptive to, and gain experiential benefit from specific therapeutic mechanisms and not others. This suggests that the effectiveness of music therapy can be significantly enhanced if, prior to music therapeutic engagement, a discussion takes place between the therapist and client about what the client would like to get out of the therapeutic encounter. In other words, it would be beneficial to establish patient’s need and preferences at the outset. However, a level of flexibility is also required in relation to the therapeutic process, with the music therapist continually observing and making clinical judgements as therapy progresses.

Another important issue raised by the research related to the articulation between contextual mechanisms and people’s responses. The pertinent contextual mechanism here is resources. Typically, hospices are small institutions, often at least partially reliant on charitable donations, with limited financial means. This would indicate that, in many cases, their capacity to fund music therapy will be limited, which in turn will tend to limit the availability of music therapists. Conversely, participants in this study suggested that a flexible music therapy regimen that was able to engage with patients at times when they were most able and receptive would enhance effectiveness. Such flexibility is resource-demanding. This tension between the inflexibility generated by resource limitations and the flexibility required to enhance therapeutic effectiveness suggests that imaginative responses are required to strike an appropriate balance. We suggest that there is a need for further research to establish at which points in the daily cycle and the timetable of care activities patients would be most able and receptive to benefit from music therapy. Such knowledge might allow music therapy to be targeted more effectively.

In conclusion, this research provides important pieces of the explanatory jigsaw that consists of establishing if music therapy is effective, how it is effective, in what circumstances, and for whom. While providing positive data in relation to the latter three considerations, and notwithstanding its demonstration that many of the participants perceived it as a beneficial therapy, the study was not designed to establish music therapy’s clinical effectiveness. That requires testing in a randomised trial, preferably across multiple sites to enhance generalisability, which has hitherto not been conducted. It is for this reason that we advocate that the next important step in establishing the effectiveness of music therapy in improving the quality of life of those receiving end-of-life care is to evaluate it via a pragmatic RCT.

## Additional files

Additional file 1: Focus Group Schedule for Practitioners. (DOCX 14 kb)

Additional file 2: Interview Schedule for Music Therapist. (DOC 47 kb)

## Abbreviations

AHP: Allied health professional
HCP: health care practitioner
MQOL: McGill quality of life questionnaire
MRC: Medical research council
NICE: National institute for health and care excellence
ORECNI: Office of research ethics committee Northern Ireland
RCT: Randomised controlled trial
UK: United Kingdom
