# Supplementary material for: A critical realist evaluation of a music therapy intervention in palliative care
Source: BMC Palliat Care. 2017 Dec 8;16:70. doi: 10.1186/s12904-017-0253-5 (PMC5723094; doi:10.1186/s12904-017-0253-5)
Supplement: Supplementary file 1 — Focus Group Schedule for Practitioners. (DOCX 14 kb) [file 12904_2017_253_MOESM1_ESM.docx]

**Focus Group Schedule for Practitioners**

**Music Therapy for Palliative Care Patients.**

Before beginning, go over the pre-interview brief with participants reminding them of confidentiality unless the researcher suspects mal-practice, which has not been declared to the appropriate agencies.

**Introduction questions relating to ‘what works’?**

1. To begin, could you describe any knowledge you had of music therapy prior to this intervention?

2. What is your understanding of the aim of music therapy in palliative care?

3. What is your understanding of how music therapy works for palliative care patients?

4. From your experience of the intervention for your patient(s) do you think music therapy works?

5. If yes, how do you think it works?

**Questions relating to ‘for whom’?**

6. Are there particular types of patient that may benefit from music therapy more than others?

7. Are there particular types of family/carers that may benefit from music therapy more than others?

8. Do you think music therapy has had any impact on you on a professional or personal level?

- If yes, why and how?

9. Do you think music therapy has had any impact on the hospice setting?

- If yes, why and how?

**Questions relating to ‘in what context’?**

10. Did you refer any of your patients to the Music Therapy study?

- If yes, why?
- If no, why?

11. What factors within the palliative care setting promote the use and effectiveness of music therapy?

- E.g. quality of support within the organisation for music therapy

12. What factors within the palliative care setting hinder the use and effectiveness of music therapy?

- E.g. funding
- Are there any issues around the sustained implementation of music therapy?
- Lack of understanding about what it involves
