# Supplementary material for: A critical realist evaluation of a music therapy intervention in palliative care
Source: BMC Palliat Care. 2017 Dec 8;16:70. doi: 10.1186/s12904-017-0253-5 (PMC5723094; doi:10.1186/s12904-017-0253-5)
Supplement: Supplementary file 2 — Interview Schedule for Music Therapist. (DOC 47 kb) [file 12904_2017_253_MOESM2_ESM.doc]

**Interview Schedule for Music Therapist**

**Music Therapy for Palliative Care Patients.**

**Introduction questions relating to therapeutic mechanisms ‘what works’?**

1. To begin, could you describe your role as a music therapist in relation to palliative/end-of-life care?

- Have you done much work in this area?
- What are the key goals of music therapy for a palliative/end-of-life population (desired outcomes)?

I’m now going to present theories of how music therapy works drawn from the literature. Could you please tell me your thoughts in relation to each theory? For example, do you agree with the theory or do you think it needs refined?

**Supportive (physical and psychological domain)**

- Gate control theory of pain. Music therapy acts as a distraction from physical and psychological suffering. Needs to influence attention, affect (evoking happier memories, improving mood and reducing anxiety), and interpretation of situation (reframe their interpretation of their situation from seeing themselves as a sick/dying patient to believing that they are an empowered individual who still has experiences to enjoy and leave behind for others).

**Communicative/Expressive (Emotional Domain**)

- Cathartic influence (bringing relief from repressed emotions and release of frustrations about their situation).
- Renewed connection with family/friends facilitated by music therapy. Safe channel for emotional expression and dialogue about spiritual conflicts.
- Less anxiety provoking than direct verbal communication.
- Therapeutic songwriting can help patients communicate thoughts and feelings in a creative, safer way.
- Musical improvisation can also help patients identify painful emotions, enabling them to communicate repressed thoughts and feelings aided by the therapist.

**Transformative (Spiritual/Existential Domain)**

- Search for meaning - integral connection between music and emotions means that listening to familiar songs and/or songwriting supports and provides a safe medium for reminiscing, exploring, and expressing feelings in palliative care patients.
- Transcendence – rising above illness and suffering through having fun with the music. Through a process of cognitive reframing, the patient can move from the perception of themselves as a sick, dying patient to that of an empowered individual who still has experiences to enjoy and leave behind for others.
- Legacy work - reduced existential anxiety by enabling patients to produce a lasting legacy in the form of songs they create as a gift for their loved ones. Having something of themselves to leave behind in the form of songs expressing their values, beliefs, and their life’s lessons provides patients with a sense of completion. Comfort to family members through sense of continued connection.

**Social Domain**

- Strengthening social bonds with loved ones
- Creates sense of community within setting – indirect benefit to staff (feel setting humanised which lifts everyone’s mood) and patients (staff able to provide better care).

**Questions relating to agency ‘for whom’?**

1. Are there particular types of patient that may benefit from music therapy more than others?
2. Are there particular types of family/carers that may benefit from music therapy more than others?
3. Do you think music therapy has had any impact on hospice staff?

- If yes, why and how?

**Questions relating to what helped or hindered music therapy achieve its desired outcomes ‘in what context’?**

11. What factors within the palliative care setting promote the use and effectiveness of music therapy?

- E.g. quality of support within the organisation for music therapy

12. What factors within the palliative care setting hinder the use and effectiveness of music therapy?

- E.g. funding
- Are there any issues around the sustained implementation of music therapy?
- Lack of understanding about what it involves
